# Structural basis for antibody binding to adenylate cyclase toxin reveals RTX linkers as neutralization-sensitive epitopes

**DOI:** 10.1371/journal.ppat.1009920

**Published:** 2021-09-21

**Authors:** Jory A. Goldsmith, Andrea M. DiVenere, Jennifer A. Maynard, Jason S. McLellan

**Affiliations:** 1 Department of Molecular Biosciences, The University of Texas at Austin, Austin, Texas, United States of America; 2 Department of Chemical Engineering, University of Texas at Austin, Austin, Texas, United States of America; Deutsches Elektronen-Synchrotron, GERMANY

## Abstract

RTX leukotoxins are a diverse family of prokaryotic virulence factors that are secreted by the type 1 secretion system (T1SS) and target leukocytes to subvert host defenses. T1SS substrates all contain a C-terminal RTX domain that mediates recruitment to the T1SS and drives secretion via a Brownian ratchet mechanism. Neutralizing antibodies against the *Bordetella pertussis* adenylate cyclase toxin, an RTX leukotoxin essential for *B*. *pertussis* colonization, have been shown to target the RTX domain and prevent binding to the α_M_β_2_ integrin receptor. Knowledge of the mechanisms by which antibodies bind and neutralize RTX leukotoxins is required to inform structure-based design of bacterial vaccines, however, no structural data are available for antibody binding to any T1SS substrate. Here, we determine the crystal structure of an engineered RTX domain fragment containing the α_M_β_2_-binding site bound to two neutralizing antibodies. Notably, the receptor-blocking antibodies bind to the linker regions of RTX blocks I–III, suggesting they are key neutralization-sensitive sites within the RTX domain and are likely involved in binding the α_M_β_2_ receptor. As the engineered RTX fragment contained these key epitopes, we assessed its immunogenicity in mice and showed that it elicits similar neutralizing antibody titers to the full RTX domain. The results from these studies will support the development of bacterial vaccines targeting RTX leukotoxins, as well as next-generation *B*. *pertussis* vaccines.

## Author summary

Diverse bacterial pathogens use the type 1 secretion system (T1SS) to secrete RTX leukotoxins, which target host leukocytes during infection. T1SS substrates all contain a repetitive C-terminal ‘RTX’ domain that adopts a characteristic β-roll fold and is involved in secretion. Notably, The RTX domain of *Bordetella pertussis* adenylate cyclase toxin (ACT) mediates leukocyte targeting via binding to the α_M_β_2_ integrin receptor, and antibodies that block receptor binding neutralize toxin activity. However, ACT also contains multiple non-neutralizing epitopes, and precise knowledge of the sites targeted by neutralizing antibodies is desirable for vaccine design. Here we determine the crystal structure of an ACT fragment in complex with two neutralizing antibodies and define the key neutralization-sensitive sites within the RTX domain. This first structure of a heterotypic protein–protein interaction formed by an RTX domain suggests the linker regions between β-roll segments engage binding partners.

## Introduction

Since the 1990s, outbreaks of pertussis have occurred in populations with significant vaccination coverage[1–7]. The observation of waning immunity post-vaccination[8–11], as well as the discovery that the acellular vaccines do not prevent transmission in nonhuman primates[12], suggest that current acellular vaccines may provide inadequate protection. In addition, circulating pertussis strains show signs of adaptation to acellular vaccine antigens, such as the widespread loss of the virulence factor pertactin[13–17], or promoter mutations that boost pertussis toxin expression[18]. One strategy for the improvement of next-generation acellular vaccines is to target the virulence factors most essential for *B*. *pertussis* colonization, as well as to optimize the presentation of their most vulnerable epitopes on vaccine antigens. The adenylate cyclase toxin (ACT) is a promising vaccine antigen as it has been shown to be essential for lung colonization in mouse intranasal models[19,20], is a protective vaccine antigen in mice, and polyclonal anti-ACT sera protect mice from *B*. *pertussis* challenge[21].

ACT belongs to the Repeats-in-ToXin (RTX) family of proteins, which are the substrates of the bacterial type I secretion system (T1SS)[22,23]. T1SS substrates include secreted toxins, proteases, lipases and adhesins[24–28]. The characteristic feature of RTX proteins is a C-terminal RTX domain containing tandem repeats of a nine-residue motif, X(H)XGGXGXD (H = hydrophobic), with the X(H)X forming a 3-residue β-strand and the GGXGXD forming a 6-residue Ca^2+^-binding turn. The tandem repetition of these 9-residue units forms a β-roll structure that is a sandwich of two parallel β-sheets, with most turns binding a Ca^2+^ ion. In the absence of Ca^2+^, the RTX domain exists in an intrinsically disordered state[29]. The primary function of the RTX domain in proteins of this family is to drive secretion across a Ca^2+^ gradient, with Ca^2+^-driven folding on the extracellular side of the T1SS preventing re-entry into the channel and therefore favoring directional transport out of the cell[30]. ACT has a large RTX domain and contains 5 stretches of 9–11 repeats in tandem, known as RTX blocks, separated by linker sequences that do not conform to the repeat consensus. In addition, all RTX proteins contain a conserved C-terminal capping structure that harbors the secretion signal for T1SS recruitment. Upon recruitment, the C-terminus is transported through the T1SS and secretion proceeds from the C-terminus to the N-terminus. For ACT, it has been shown that this capping structure is essential for folding of the entire RTX domain, as well as for toxin activity[31]. NMR spectroscopic analysis of a C-terminal ACT fragment consisting of RTX block V and the C-terminal cap in the presence of increasing concentrations of Ca^2+^ suggests that folding proceeds directionally from the C-terminus starting with the cap[30]. The cap is likely required to nucleate folding of the β-roll by limiting the conformational entropy of the C-terminus[32].

ACT is related to the RTX leukotoxins, such as *E*. *coli* hemolysin A, which are secreted by bacterial pathogens to subvert mammalian host defenses by targeting leukocytes. ACT contains an N-terminal adenylate cyclase enzyme and a large internal hydrophobic region that inserts into host cell membranes. Upon insertion, the adenylate cyclase enzyme is translocated through the host membrane by a poorly understood mechanism, resulting in aberrantly high cAMP levels that disrupt leukocyte functions and can result in cell death. ACT has been shown to use the α_M_β_2_ integrin as a receptor, with this interaction being required for intoxication of macrophage J774A.1 cells[33] as well as CHO cells recombinantly expressing α_M_β_2_[34]. Interestingly, ACT has adapted its RTX domain to serve a receptor-binding role, with the binding site localized within residues 1166–1287 (spanning RTX blocks I–III)[33].

Previous characterization of isolated monoclonal antibodies obtained from mice immunized with ACT showed that most antibodies bound the RTX domain, suggesting it is immunodominant[35]. In this previous study, anti-RTX antibodies were divided into six competition groups, with only two of those groups containing neutralizing antibodies. Notably, the antibodies from the two neutralizing competition groups block receptor binding, supporting the feasibility of developing vaccine antigens that target the ACT/α_M_β_2_ interaction. Consistent with the putative α_M_β_2_ binding site in blocks I–III, the epitopes of M2B10 and M1H5 have been mapped to blocks I and II, and blocks II and III, respectively[36]. However, the structural basis for engagement of the RTX toxins by neutralizing antibodies is not known.

Here, we determine the crystal structure of the receptor-binding blocks I–III of the *B*. *pertussis* adenylate cyclase toxin in complex with neutralizing antibodies that prevent binding to α_M_β_2_. To generate a receptor-binding domain construct amenable to crystallography, we engineered a minimal variant of the RTX domain with blocks I–III of the RTX domain fused to the block V capping structure required for RTX folding. As our designed RTX fragment contained the two neutralization-sensitive epitopes, we assessed its ability to elicit neutralizing anti-ACT antibodies. These findings define key neutralizing epitopes on the *B*. *pertussis* ACT in atomic detail, providing critical tools for the structure-based design of immunogens targeting this virulence factor for next-generation whooping cough vaccines.

## Results

### Design of cap-stabilized RTX domain fragments containing blocks I–III

To define the neutralization-sensitive epitopes on the ACT RTX domain bound by antibodies M2B10 and M1H5 at high resolution, we sought to crystallize complexes of the corresponding antigen-binding fragments (Fabs) bound to the RTX domain. We attempted crystallization of either one or both Fabs bound to RTX751 (ACT residues 751–1706) or RTX985 (ACT residues 985–1706)[35]. However, none of these combinations yielded well-diffracting crystals.

**Fig 1:**
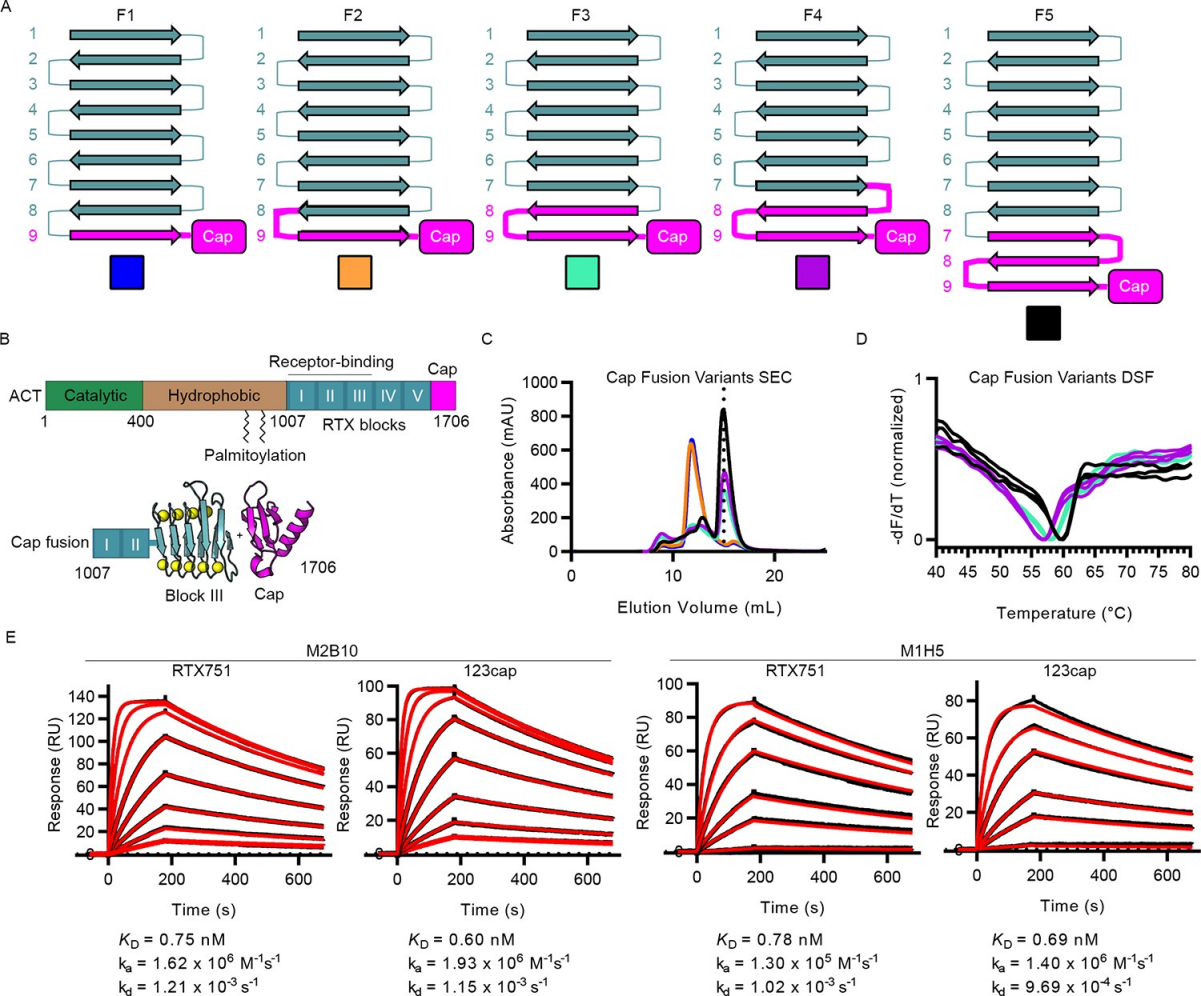
Design and characterization of cap-stabilized RTX domain fragments containing blocks I–III. (A) Design scheme of fusions between block III and block V cap fragment. RTX repeats from block III and V are represented in terms of their β-strands, numbered by RTX repeat within each RTX block, and their connecting segments, which are mostly Ca^2+^-binding turns but can contain loop insertions. For each variant, the strands and connecting segments from block III are shown in teal, and those from block V are shown in magenta. (B) Schematic of the ACT gene showing the catalytic domain (green), hydrophobic segment (brown), palmitoylation sites, RTX domain with RTX blocks (teal) and C-terminal cap (magenta). The scheme is shown for fusing the C-terminal cap to a fragment containing RTX blocks I-III, with block III and the C-terminal cap shown in 3D cartoon representation. (C) Size-exclusion chromatography elution profiles for purified cap fusion variants, with colors corresponding to the boxes in A. The dotted line represents the peak elution volume that is consistent with monomeric 123cap. (D) Differential scanning fluorimetry melting profiles for F3, F4, and F5, with data shown as the negative first derivative of the fluorescence intensity. Colors correspond to the boxes in A. (E) Surface plasmon resonance kinetic measurements of M2B10 and M1H5 Fab binding to RTX751 and 123cap. Measured signal for each Fab concentration is shown as a black line, and kinetic fits are shown as red lines. Fit kinetic parameters and equilibrium dissociation constants are shown for each interaction under the corresponding concentration series.

Given the difficulty in crystallizing the full RTX domain, a smaller RTX fragment containing only RTX blocks I–III was desired. Blocks I–III contain the putative α_M_β_2_ binding site and the epitopes of M2B10 and M1H5[33,36]. However, the RTX domain does not tolerate C-terminal truncations, as this removes the C-terminal capping structure required for folding[30]. We therefore sought to design a minimal RTX variant containing only blocks I–III fused at the C-terminus to the block V capping structure to nucleate folding. We reasoned that fusing the capping structure to an internal RTX block would be relatively straightforward provided that the sequence of 3-strand β-sheets and 6-residue Ca^2+^-binding turns would remain unperturbed. Using this rationale, different graft points were chosen to yield 5 fusion designs, each with residues from the block V cap fragment fused in-frame with the block III RTX repeat (Fig 1A and S1 Table). Designs containing differing numbers of repeats from the C-terminus of block V were chosen as it was not known whether the nucleation function of the cap depends on interactions with the specific amino acids in the adjacent repeats being nucleated. Each design was expressed in *E*. *coli* and subjected to size-exclusion chromatography (SEC) to assess oligomeric state and monodispersity (Fig 1C). Although soluble protein was obtained for each variant, F1 and F2 expressed only as a higher order oligomer. Variants F3, F4 and F5, which exhibited SEC peaks at the expected elution volume of a monomer, each contained at least the final two repeats from block V. By contrast, the aggregated F1 and F2 only contained the final repeat. F5, the only variant containing the final 3 repeats from block V, had the highest yield of monomeric protein. In addition, differential scanning fluorimetry showed that F5 had the highest melting temperature of the monomeric variants (60°C) (Fig 1D). Thus, we selected F5 for structural studies, and renamed it ‘123cap’. The trend of increased stability and expression with increasing amounts of the block V C-terminal RTX repeats suggests that the C-terminus of block V may be required for the nucleation provided by the C-terminal cap.

To determine whether the folded conformation of 123cap accurately recapitulates the antibody-binding sites as presented by full RTX, we measured the binding kinetics and affinity of the neutralizing antibodies M2B10 and M1H5 for both RTX751 and 123cap using surface plasmon resonance. The affinities of M2B10 for RTX751 and 123cap were 0.75 nM and 0.60 nM respectively. Similarly, the affinities of M1H5 for RTX751 and for 123cap were 0.8 nM and 0.7 nM respectively. The similar affinity of the neutralizing antibodies for the two antigens, in conjunction with similar monomeric SEC and thermal unfolding profiles, suggest that 123cap retains a similar structure as the full RTX domain.

### Crystal structure of 123cap bound to neutralizing antibodies

To define the neutralization-sensitive epitopes of M2B10 and M1H5 at an atomic level, and to characterize the overall structure of the ACT receptor-binding domain, we determined a 2.6 Å resolution crystal structure of 123cap in complex with both M2B10 and M1H5 Fabs. The ternary complex crystallized in space group *P*2_1_2_1_2_1_ and contained 1 complex per asymmetric unit. The structure was solved by molecular replacement and refined to an *R*_work_/*R*_free_ of 19.5%/21.8% (S2 Table), using the crystal structure of RTX block V and the C-terminal cap (PDB ID: 5CVW) to generate search models for RTX blocks I-III and the cap, and using Fab models for M2B10 and M1H5.

**Fig 2:**
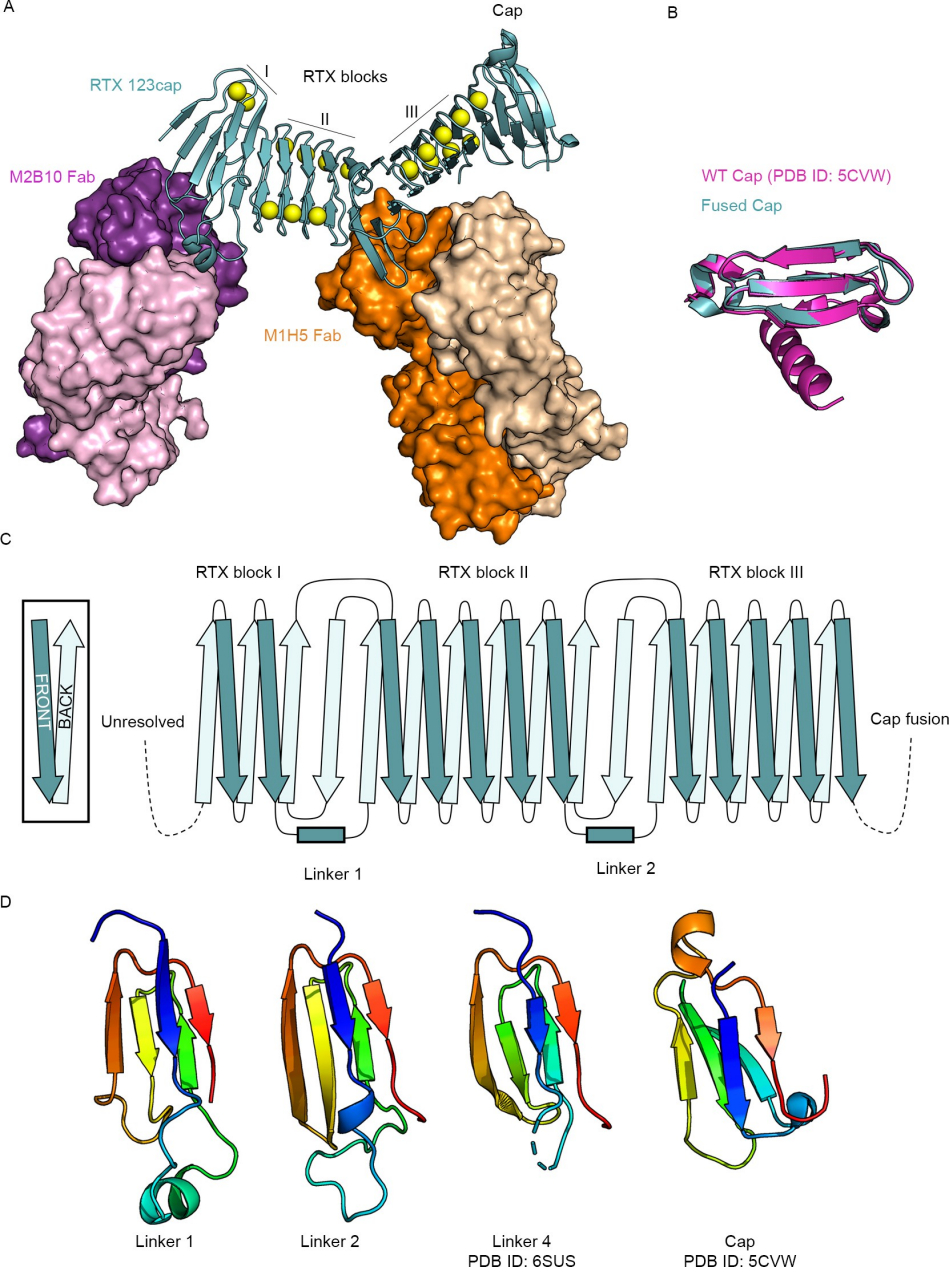
Crystal structure of 123cap bound to the receptor-blocking antibodies M2B10 and M1H5. (A) Overall structure of 123cap in complex with M2B10 and M1H5 Fabs. Ca^2+^ ions are shown as yellow spheres. (B) Structural alignment of the capping structure of 123cap with the WT capping structure from the crystal structure of block V (PDB ID: 5CVW), with the WT cap shown in magenta and the fused cap shown in teal. (C) Topology diagram of blocks I–III from 123cap, including the topology of linker 1 and linker 2. (D) Structure of L1, L2, L4 and the region from the capping structure with the same topological motif. Each is colored as an N to C rainbow (blue to red) to show the path of the mainchain.

The structure revealed that blocks I–III of the RTX domain form an elongated β-roll with a continuous hydrophobic core, as was observed for the recently determined structure of blocks IV and V[37]. Notably, the capping structure of 123cap overlays well with the wild-type cap from the structure of block V[30], showing that the cap can be successfully grafted onto internal RTX blocks to support their proper folding (Fig 2B). However, the sequence expected to be the 8^th^ repeat of block III, consisting of residues 1325–1333[31], was excluded from the β-roll and is present as a loop between repeat 7 of block III and repeat 7 of block V (Fig 1A, F5). This is likely an artefact of the graft between blocks III and V and suggests an improved 123cap could be generated by removing repeat 7 or 8 of block III to remove the extra loop. In addition, N-terminal residues 1007–1055 were unresolved in the structure (Fig 2C), which is consistent with the N-terminal RTX domain also requiring entropic stabilization[32], but may also be due to inherent flexibility of this region.

**Fig 3:**
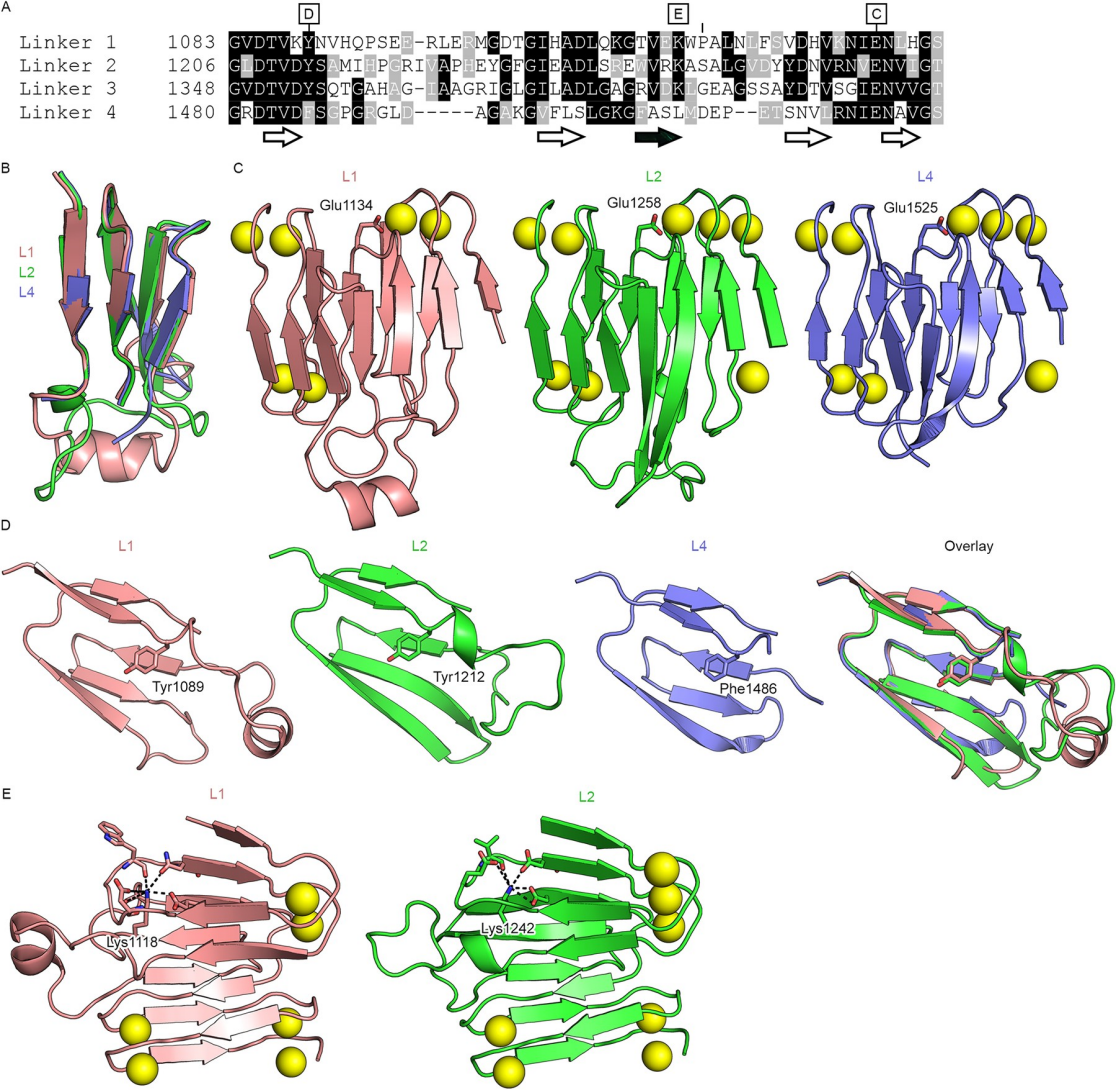
The ACT inter-block linkers are conserved modules. (A) Sequence alignment of L1, L2, L3, and L4 from *B*. *pertussis* ACT. The locations of the core β-sheets are shown below the alignment, with the antiparallel β-strand shown with a black fill. Letters above the alignment denote the subsequent panels that highlight the specified residue. (B) Structural alignment of L1, L2 and L4 (L4 from PDB ID: 6SUS). (C) Conserved Ca^2+^-binding glutamate at the C-termini of L1, L2, and L4. (D) Conserved core tyrosine/phenylalanine at the N-termini of L1, L2, and L4. (E) Partially conserved buried lysine residue in L1 and L2.

Notably, the linker regions between blocks I and II (L1) and between blocks II and III (L2) both connect their adjacent RTX blocks using the same topological motif as L4, which connects blocks IV and V (Fig 2D)[37]. In the RTX blocks, where the repeats conform to the RTX consensus, there are an equal number of β-strands on each face of the β-roll. The linker motif, in contrast, introduces an additional antiparallel strand onto one face of the β-roll, and therefore introduces a kink into the RTX domain between the two connected blocks (Fig 1A and 1B). It was previously noted that the L4 topological motif was also present within the C-terminal cap[37]. However, structural alignment of L4 and the cap motif shows that they have differing conformations. By contrast, the core β-sheets of the L1, L2 and L4 linkers have nearly identical mainchain conformations (Fig 3B). Previously, RTX repeats were annotated based on the extent to which they conform to the RTX consensus, and the linkers were defined as the stretches between blocks of RTX repeats that did not recognizably conform[31]. However, when defined by the conserved fold, the boundaries of the linkers differ from this sequence-based annotation. Sequence alignment of the regions defined by the conserved structural motif reveals sequence homology between L1, L2 and L4 (Fig 3A). The alignment also shows that L3, whose structure is not known, contains the same motif, and that L1, L2 and L3 are more similar to each other than they are to L4 (Fig 3A). This suggests that the long RTX domain of ACT may have evolved via duplication of modules composed of one RTX block and one linker. Notably, each linker contains a conserved tyrosine/phenylalanine (Tyr1089 in L1, Tyr1212 in L2, Tyr1354 in L3, Phe1486 in L4, Fig 3D) at the N-terminus that packs into the hydrophobic core against the core-facing residue of the antiparallel β-strand, which is a valine in L1, L2 and L3. In addition, the linkers all contain a conserved (I/V)EN near the C-terminus, whereby the glutamate (Glu1134 in L1, Glu1258 in L2, Glu1525 in L4) coordinates the Ca^2+^ ion from the RTX block N-terminal to the linker in place of a canonical Ca^2+^-binding turn (Fig 3C). Interestingly, a lysine that is conserved in the L1, L2 and L3 sequences is seen to be buried in the structures of L1 and L2 (Lys1118 and Lys1242, respectively), with its terminal ε-amino group occupying the position between two Ca^2+^-binding turns that would typically be occupied by a Ca^2+^ ion (Fig 3E). Overall, these conserved features delineate the inter-block linkers as distinct modules.

**Fig 4:**
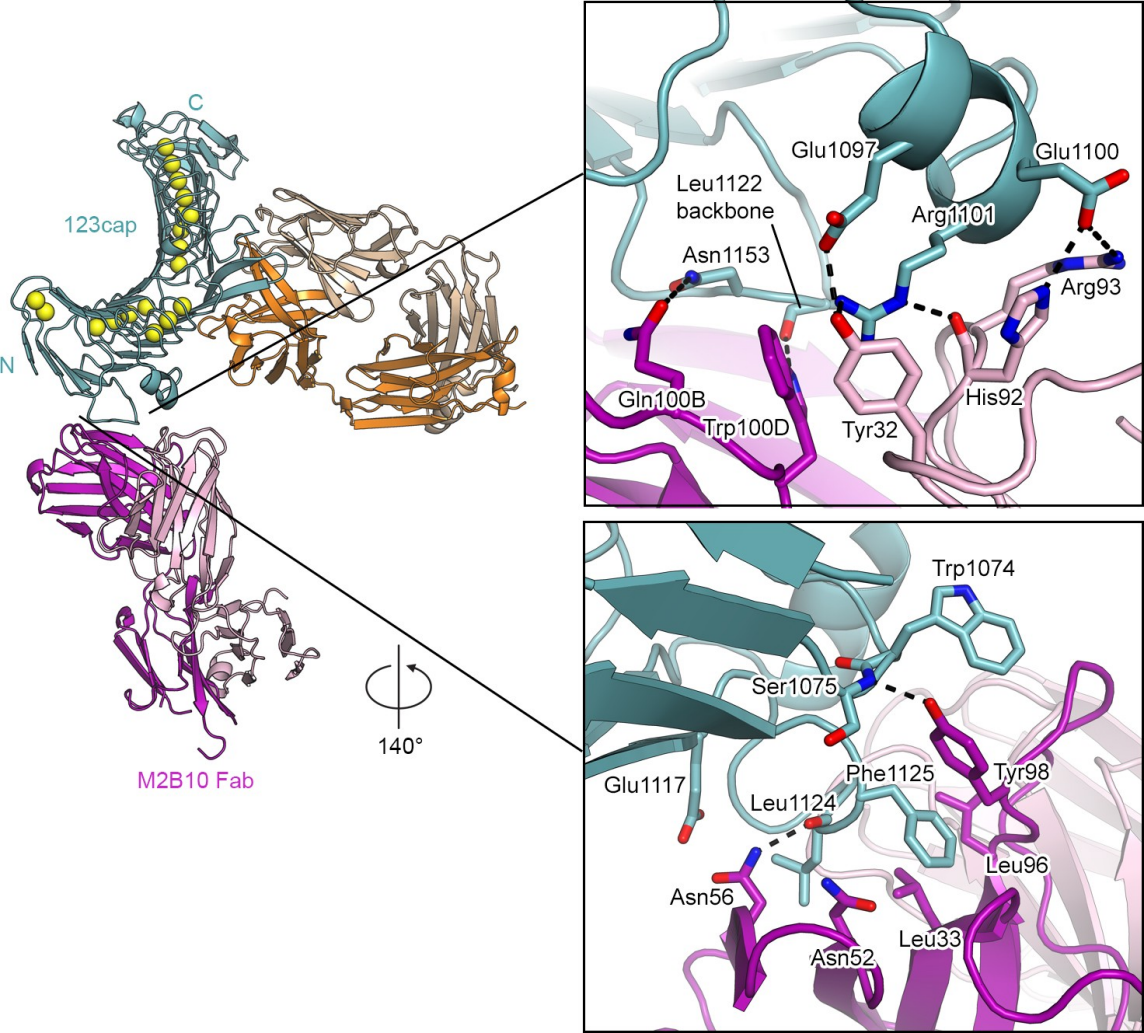
Interactions formed by M2B10 with linker 1 of the RTX domain. Structures are shown as ribbon representation with select residues shown as sticks, with nitrogens colored blue, oxygens colored red, and sulfur colored yellow. Calcium is represented as yellow spheres.

M2B10 engages L1 of the RTX domain using both its heavy and light chains, with the light chain contacting the short α-helix in the first loop of L1 (Fig 4). Specifically, His92 and Arg93 of the CDRL3 form salt bridges with the sidechain of Glu1100 of the L1 helix (Fig 4, top). In addition, Tyr32 of the CDRL1 as well as Trp100D of the CDRH3 form π-cation interactions with Arg1101 of the L1 helix. The interaction of the light chain with the L1 helix is also stabilized by hydrogen bonds between the CDRL3 His92 backbone and the sidechain of Arg1101, as well as between the CDRL1 Tyr32 and Glu1097. Additionally, the sidechains of Gln100B and Trp100D in the CDRH3 form hydrogen bonds with the loop in L1 that follows the antiparallel β-strand. (Fig 4, top). This loop also contains Phe1125, which inserts into a hydrophobic pocket formed by Leu96 and Tyr98 in the CDRH3, as well as Leu32 of the CDRH1. The Asn56 sidechain in the CDRH2 also hydrogen bonds with the Leu1124 backbone in this same loop in L1. Notably, Tyr98 in the CDRH3 stacks against Trp1074 of RTX block I, and also forms a hydrogen bond with the mainchain nitrogen of Ser1075 using its sidechain hydroxyl. The contacts formed by Tyr98 with Trp1074 and Ser1075 are the only ones formed by M2B10 with the repeat region of the RTX domain (Fig 4, bottom).

**Fig 5:**
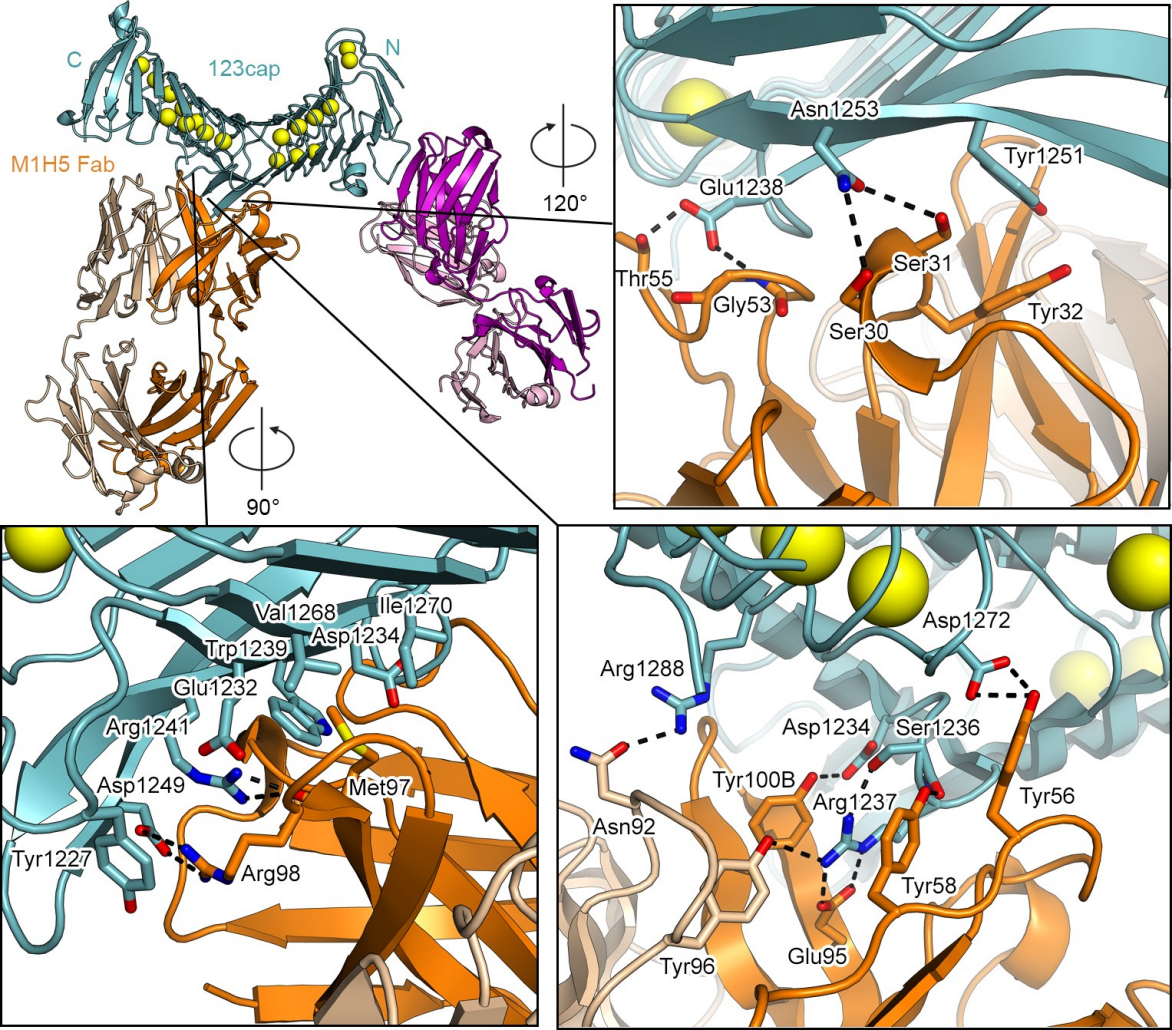
Interactions formed by M1H5 with linker 2 of the RTX domain. Structures are shown as ribbon representation with select residues shown as sticks, with nitrogens colored blue, oxygens colored red, and sulfur colored yellow. Calcium is represented as yellow spheres.

M1H5 contacts L2 almost exclusively using its heavy chain, with buried surface areas of 816 Å^2^ and 146 Å^2^ for the heavy and light chain interfaces, respectively. Tyr100B of the CDRH3 forms a hydrogen bond with Asp1234, a residue that has been implicated in α_M_β_2_ binding[34]. Arg1237 in L2 is a key residue in the M1H5 interface. The Arg1237 guanidinium group is sandwiched on either side by Tyr100B in the CDRH3 and Tyr58 in the CDRH2, forming π-cation interactions. Arg1237 also forms a salt bridge with Glu95 in the CDRH3, and hydrogen bonds to Tyr96 in the CDRL3. Arg1288 and Asp1272, both in RTX block III, form hydrogen bonds with Asn92 of the CDRL3 and Tyr56 of the CDRH2, respectively, which are the only interactions formed by M1H5 with the RTX repeat region (Fig 5, bottom right). Arg98 of the CDRH3 forms a salt bridge with Asp1249 of L2 and simultaneously forms a π-cation interaction with Tyr1227. CDRH3 Met97 inserts its sidechain into a hydrophobic pocket in L2 formed by Trp1239, Val1268, Asp1234, Ile1270, and Glu1232, and the backbone carbonyl of Met97 forms a hydrogen bond with the sidechain of Arg1241 (Fig 5, bottom left). In addition, CDRH1 Tyr32 forms a π-π stacking interaction with Tyr1251 in L2. Lastly, Glu1238 forms hydrogen bonds with CDRH2 Gly53 and Thr55 and Asn1253 forms hydrogen bonds with CDRH1 Ser30 and Ser31 (Fig 5, top right).

These data suggest that L1 and L2 are used by ACT to engage the α_M_β_2_ receptor, as binding of either antibody prevents this interaction[35]. In addition, they also suggest that the linker regions may be particularly immunogenic, with L1 and L2 likely representing the most critical epitopes to include in an RTX-based vaccine antigen.

### Immunogenicity of 123cap and RTX751 in mice

**Fig 6:**
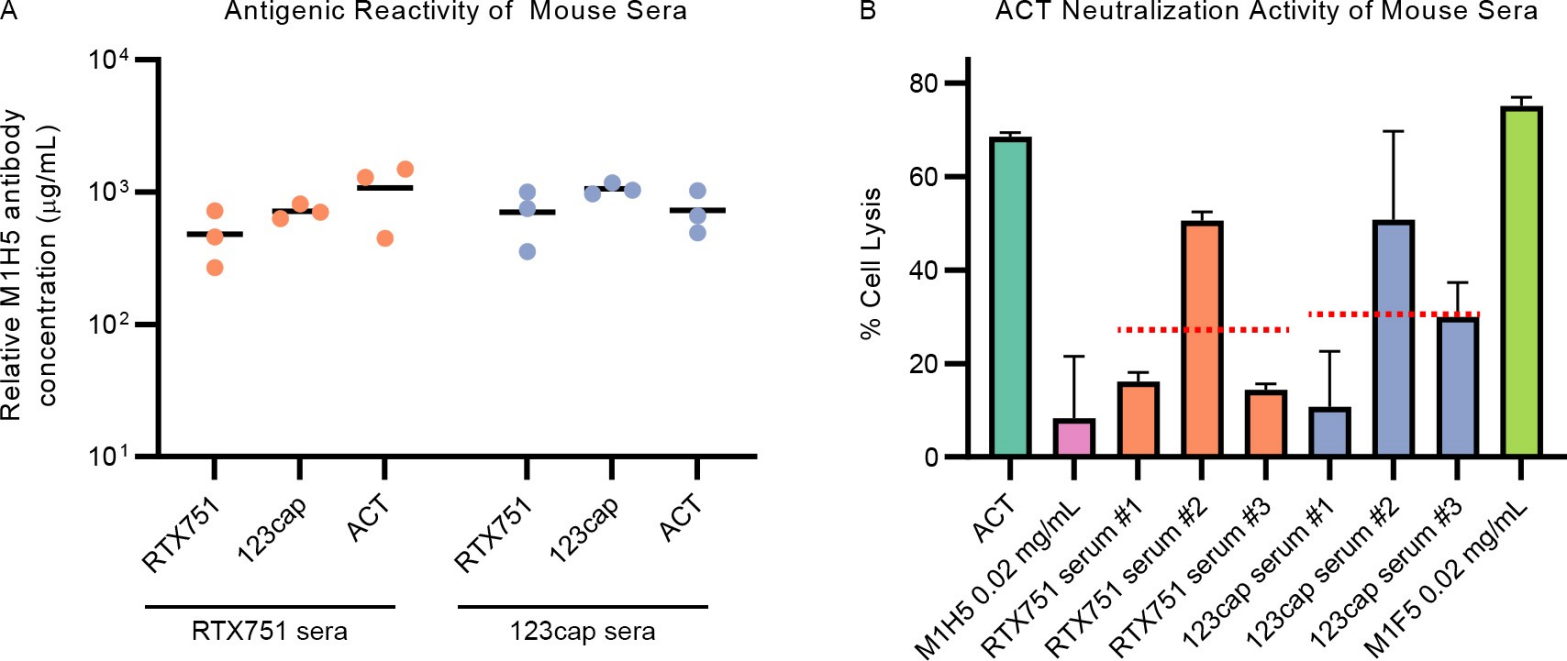
RTX751 and 123cap elicit similar neutralizing antibody responses. (A) Mouse serum titers against RTX751, 123cap and ACT after immunizing and boosting with either RTX751 or 123cap and Freund’s adjuvant. Each data point represents one mouse, with titers measured by ELISA using the murine M1H5 antibody as a standard. The x-axis tick labels define the antigen of reactivity. (B) Toxin neutralization assay with 0.5 ug/mL ACT added to J774A.1 cells in the presence of a 30-fold molar excess of a neutralizing anti-ACT antibody (M1H5), non-neutralizing anti-ACT antibody (M1F5) or immune sera against 123cap or RTX751 (1:250 dilution, previously determined to be in the dose-response range; individual mice labeled #1, #2, #3). Each bar represents the mean of 2 independent assays, with error bars representing the standard error of the mean, and with each assay containing 3 technical replicates. The mean cell lysis value for sera resulting from RTX751 and 123cap immunization are shown as dashed red lines.

As 123cap contains both the M2B10 and M1H5 epitopes, it could potentially serve as an immunogen that focuses responses to L1 and L2 while avoiding elicitation of non-neutralizing antibodies. Therefore, we performed mouse immunization experiments to characterize the immunogenicity of 123cap in comparison to full-length ACT and RTX751. Five-week-old Balb/c mice were immunized with ACT, RTX751 or 123cap and Freund’s complete adjuvant, boosted four weeks later with the same antigen dosage and Freund’s incomplete adjuvant, with serum harvested two weeks after boosting. Binding of total serum IgG to RTX variants was measured, with binding titers against ACT, RTX751 and 123cap being similar upon immunization with either RTX751 or 123cap (Fig 6A). Assessment of the ability of vaccine-induced sera to neutralize ACT in a cell-lysis assay showed that sera resulting from immunization with both RTX751 and 123cap afforded similar decreases in cell lysis (Fig 6B). These results suggest that 123cap retains the relevant neutralization-sensitive epitopes from the full RTX domain.

## Discussion

ACT is an attractive target for next-generation vaccines given its essential role in establishing initial *B*. *pertussis* colonization in mouse intranasal challenge models[19,20]. Previous mouse immunization experiments showed that the RTX domain of ACT is immunodominant and elicits neutralizing antibodies[35], suggesting it should be a focus of vaccine design efforts. The data presented here suggest that the linker regions between RTX blocks may be the immunodominant sites within the RTX domain, although the sites bound by the four non-neutralizing RTX competition groups are not yet known. However, it is intuitive that the RTX domain would engage in protein-protein interactions using its inter-block linkers, as the β-roll structure that results from tandem repeats of the consensus is extremely flat and does not have an optimal surface for the formation of an extensive binding interface. The deviation from the flat β-roll and presence of longer loops allow the linker regions to engage antibodies, and likely the α_M_β_2_ receptor. Specifically, the structure of 123cap bound to the neutralizing antibodies M2B10 and M1H5—representing the two neutralizing competition groups of RTX domain antibodies—shows that the two corresponding neutralizing sites are L1 and L2. Therefore, a focus of vaccine design could be the optimal presentation of L1 and L2, with the goal of eliciting M1H5-like and M2B10-like antibodies.

In addition, the ability of M2B10 and M1H5 to inhibit α_M_β_2_ binding[35] suggests that L1 and L2 are involved in engaging α_M_β_2_. Notably, L1 and L2 reside within the previously identified putative receptor-binding site spanning ACT residues 1166–1287[33]. The double mutant D1232A/D1234A and the triple mutant D1193A/D1194A/E1195A evaluated in that study are both deficient in binding cells expressing α_M_β_2_ as well as in α_M_β_2_-dependent cell intoxication[34]. Asp1232 and Asp1234 are exposed in L2 and are part of the M1H5 binding interface (Fig 5), which suggests L2 contains at least part of the receptor-binding surface. Asp1193, Asp1194, and Asp1195 are not within L2 but rather the repeat immediately preceding L2, however they are only partially surface-exposed and are involved in internal hydrogen-bonding networks and Ca^2+^ coordination. Thus, it is likely that mutation of these three residues to alanine results in a local destabilization of L2, still pointing to the importance of this region in α_M_β_2_ binding. No mutations specifically localized to L1 have been identified that prevent α_M_β_2_ binding, therefore it is unclear whether the M2B10 binding site additionally represents a significant portion of the receptor-binding interface or M2B10 merely clashes sterically with the receptor. The α_M_β_2_ ectodomain is ~110 Å in its longest dimension and could accommodate an interface spanning L1 and L2, which are ~35 Å apart.

The β-roll structure formed by tandem RTX repeats contains mostly local interactions. As has been noted for repeat proteins in general, this simplifies the relationship between sequence and structure and therefore allows fusion of disparate regions into rigid units by keeping them “in-frame” with respect to the consensus sequence[38]. Here we show that the proper folding of an RTX domain fragment containing blocks I–III can be achieved by fusing it to the C-terminal cap of block V, demonstrating that this can be a successful strategy for expressing internal RTX blocks. During preparation of this manuscript, a publication from Espinosa-Vinals et al.[39] similarly showed that ACT and RTX variants truncated at residue 1294 (in block III) could be expressed and were functional when fused to residues 1562–1706, containing half of block V and the capping structure.

The homology between the linkers of the ACT RTX domain suggests that they may share a common ancestor, with the evolutionary history of ACT or one of its ancestors potentially involving repeat expansion of a single linker. The conserved features of the inter-block linkers suggest they should be relatively straightforward to incorporate into generic RTX scaffolds. This could allow for the rational design of antigens that optimally present L1 and L2 to immune cells, such as synthetic RTX proteins containing arrays of L1 and L2. In such arrays, the distance between linkers could be tuned by the number of repeats in the intervening RTX blocks and the direction of the kinks in the β-roll would depend on which face of the β-roll enters the linker motif. Linker arrays could also be used to expand the breadth of coverage by presenting linkers with polymorphisms present in different *B*. *pertussis* strains or other *Bordetella* species. In addition, it may be possible to optimize expression and stability of a generic RTX scaffold that is not evolutionarily constrained by the function of the overall toxin. Previously, an ankyrin repeat protein was designed by having the repeat regions conform strictly to the consensus, and it was found that this protein was more stable than naturally occurring ankyrin repeats[40]. Similar results were obtained by designing a consensus tetracopeptide repeat protein, with it similarly exhibiting greater stability than its natural counterpart[41]. Thus, an analogous strategy could be employed to design a stable RTX repeat scaffold to present RTX linkers.

Lastly, we show that 123cap can elicit similar levels of neutralizing antibodies to the wild-type variant RTX751, which contains the entire RTX domain. This suggests that 123cap contains most or all the neutralization-sensitive epitopes of the RTX domain, meaning 123cap or a similar immunogen could be used to focus the immune response onto L1 and L2, with the goal of eliciting neutralizing antibodies that target α_M_β_2_ binding. In the recent publication from Espinosa-Vinals et al.[39], an RTX variant containing only L2 and not L1 was unable to out-compete WT ACT for cell-binding, whereas all N-terminally extended variants that contained L1 were able to block ACT binding to cells. In addition, immune sera elicited by the variants containing L1 exhibited significantly greater inhibition of cell binding by ACT. The improved ability of variants containing L1 to elicit receptor-blocking antibodies is consistent with L1 being a key epitope that elicits M2B10-like antibodies.

Despite ACT being essential[20], its potential role as a protective antigen has been harder to define. Prior reports evaluated this in mouse models using ACT immunization, either alone or in combination with acellular vaccines [42–44]. While some of these reports observed significant reductions in bacterial lung colonization attributable to ACT, others did not. We hypothesize that these experiments have been complicated by heterogeneous ACT protein preparations that present neutralizing epitopes in various conformations as well as the presence of non-neutralizing epitopes that can limit protective responses. The 123cap immunogen described here will help to elucidate these issues in future work.

## Materials and methods

### Ethics statement

All animal procedures were performed in a facility accredited by the Association for Assessment and Accreditation of Laboratory Animal Care International in accordance with protocols approved by University of Texas, Austin (2018–00092) Animal Care and Use Committees and the principles outlined in the Guide for the Care and Use of Laboratory Animals.

### Protein expression and purification

To clone the RTX cap fusion variants, N-terminal and C-terminal fragments were PCR-amplified and fused by overlap PCR, before being cloned into the pET22b bacterial expression vector downstream of an N-terminal 8xHis tag and human rhinovirus (HRV) 3C protease cleavage site. RTX751 (ACT residues 751–1706) was also cloned downstream of an N-terminal 8xHis and HRV 3C protease site. RTX variants were transformed into *E*. *coli* BL21 DE3 for IPTG-inducible overexpression. Transformed cells were grown to OD_600_ = 0.6, after which 1 mM IPTG was added and cells were grown for 16 h at 16°C. Cells were then resuspended in 50 mM Tris-HCl pH 8, 200 mM NaCl, 10 mM imidazole, 2 mM CaCl_2_ and lysed via two passages through a Microfluidics LM10 Microfluidizer at 18,000 psi. Lysate was then centrifuged for 1 h at 30,000xg to clarify soluble fraction. Equilibrated HisPur Ni-NTA Resin (Thermo Fisher) was stirred with clarified lysate for 30 min., after which the flow-through was collected and the resin was washed with 50 mM Tris-HCl pH 8, 200 mM NaCl, 40 mM imidazole, 2 mM CaCl_2_. The protein was eluted with 50 mM Tris-HCl pH 8, 200 mM NaCl, 150 mM imidazole, 2 mM CaCl_2_, concentrated, and further purified by size-exclusion chromatography using a HiLoad 16/600 Superdex 200 pg column (Cytiva) and a running buffer of 20 mM Tris-HCl pH 7.5, 150 mM NaCl, 2 mM CaCl_2_. Protein used for crystallization was purified in the same fashion, but with 10 mM SrCl_2_ in the lysis, wash, and Ni-NTA elution buffers. After size-exclusion chromatography, protein was concentrated down to 1–3 mg/mL, flash-frozen using liquid N_2_, and stored at -80°C.

To allow for efficient generation of Fabs, the VH from M2B10 and M1H5 were cloned into a heavy chain expression vector containing an HRV 3C protease site between the CH1 and CH2 domains. To express M2B10 and M1H5 IgG, the heavy and light chain vectors were transiently co-transfected into FreeStyle 293-F cells (Invitrogen). After 6 days, supernatant was harvested and passed through a 0.22 μM filter. IgG was then purified from the supernatant using Protein A Agarose (ThermoFisher). Fab was produced by incubating IgG with 1:20 wt/wt HRV 3C protease for 1 h at room temperature, depleting the Fc by passing the reaction over Protein A Agarose (ThermoFisher), and subsequently separating the Fab from the HRV 3C protease by size-exclusion chromatography using a HiLoad 16/600 Superdex 200 pg column (Cytiva) and a running buffer of 20 mM Tris-HCl pH 7.5, 150 mM NaCl, 2 mM CaCl_2_. Protein was then concentrated down to 5–10 mg/mL, flash-frozen using liquid N_2_, and stored at -80°C.

Immunoprecipitation with M2B10 and M1H5 was used to purify the 123cap+M2B10 Fab+M1H5 Fab complex used for crystallography. 2-fold molar excess of each IgG was added to 123cap and incubated for 10 min. at room temperature. The binding reaction was then passed over Protein A Agarose (ThermoFisher), the resin was washed, and the ternary complex was released by rotating the resin in buffer containing 1:20 wt/wt HRV 3C protease relative to the mass of IgG overnight at 4°C. The ternary complex was then concentrated and further purified by size-exclusion chromatography using a Superdex 200 Increase 10/300 GL column (Cytiva) and a running buffer of 20 mM Tris-HCl pH 7.5, 150 mM NaCl, 2 mM CaCl_2_. After size-exclusion chromatography, the ternary complex was concentrated down to 3.5 mg/mL, flash-frozen using liquid nitrogen, and stored at -80°C.

### Differential scanning fluorimetry

RTX cap fusion variants F3, F4 and F5 were diluted to 0.25 mg/mL with buffer containing 20 mM Tris-HCl pH 7.5, 150 mM NaCl, 2 mM CaCl_2_ and a final concentration of 5X SYPRO Orange dye (diluted from a 2000X stock) in triplicate in a 96-well plate. Melting profiles were obtained using a Roche LightCycler 480 II by taking continuous fluorescence (λex = 465 nm, λem = 580 nm) with measurements at 480 nm, with a temperature range of 22°C to 95°C and a ramp rate of 4.4°C/min.

### Surface plasmon resonance

To measure the kinetics of M2B10 Fab and M1H5 Fab binding, RTX751 and 123cap with N-terminal 8xHis tags were immobilized onto an NTA sensor chip (Cytiva) in a Biacore X100 (Cytiva) using a running buffer of 10 mM HEPES pH 8.0, 150 mM NaCl, 2 mM CaCl_2_, 0.05% Tween-20. Following immobilization of ~200 RU RTX751 or ~100 RU 123cap, Fab solution (0.1 nM, 1 nM, 2 nM, 5 nM, 10 nM, and 25 nM for M1H5; 0.4 nM, 0.8 nM, 1.6 nM, 3.1 nM, 6.3 nM, 12.5 nM, 25 nM, 50 nM for M2B10) was flowed in and association was measured for 120s, with buffer being subsequently flowed in for 600s to measure dissociation. Regeneration and re-functionalization of the NTA was performed after each association and dissociation, cycle using 0.35 M ethylenediaminetetraacetic acid (EDTA) followed by 0.5 mM NiCl_2_. Global association and dissociation constants were obtained by fitting the data to a 1:1 binding model.

### X-ray crystallography

Ternary complex of M2B10 Fab+M1H5 Fab+123cap at 3 mg/mL was mixed with mother liquor (0.1 ul + 0.1 ul) containing 0.1M sodium potassium phosphate pH 6.2, 10% PEG8000, 0.2 M NaCl, 3% sucrose using an NT8 (Formulatrix), which subsequently spotted the 0.2 ul drop into an MRC2 sitting-drop crystallization tray to allow for vapor diffusion, with the well containing 60 ul of the mother liquor. A 2.6Å diffraction dataset was obtained using a crystal that was rapidly soaked in 0.1 M sodium potassium phosphate pH 6.2, 10% PEG8000, 0.2 M NaCl, 3% sucrose, 30% ethylene glycol, before being flash-frozen in liquid nitrogen. Diffraction data were collected at the SBC beamline 19ID (Advanced Photon Source, Argonne National Laboratory). Indexing and integration of the diffraction data were performed in iMOSFLM[45], before being subsequently scaled to 2.6Å and merged using AIMLESS[46]. Molecular replacement was performed with Phaser[47]. Iterative rounds of model building and refinement in Coot[48], Phenix[49], and ISOLDE[50] were then performed to arrive at a final model. Data collection and refinement statistics can be found in S2 Table.

### Mouse immunization

Five-week-old Balb/c mice were subcutaneously immunized with 10 μg of RTX751 or 123cap, or 15 μg of ACT, and Complete Freund’s Adjuvant (Sigma). Four weeks later, mice were boosted with the same amount of protein and Incomplete Freund’s Adjuvant (Sigma). Two weeks after boosting, mice were sacrificed by cardiac puncture for serum collection.

### Measurement of anti-RTX IgG titers

For serological analyses, plates were coated with 0.5–1 μg/mL antigen in HBSC (HBS + 2 mM CaCl_2_) in high protein binding 96-well plates (Corning) overnight at 4°C. Plates were blocked with 5% milk in PBS+0.1% Tween-20 (PBS-T) for one hour prior to the addition of duplicate control mouse antibody M2B10 or sera, starting at 2 mg/mL or a 1:200 dilution, respectively, and serially diluted 5-fold. Controls included the same antibody dilutions on uncoated wells and coated wells with no primary antibody. After a one-hour incubation at room temperature, plates were washed three times with PBS-T and 1:2000 anti-mouse Ig-HRP secondary (Southern Biotech) with PBS-T + 5% milk as diluent. Binding was assessed with the addition of freshly prepared 1:1 TMB (Pierce) and quenched with an equal volume of 1 N HCl. Absorbance values at 450 nm were measured using a SpectraMax M5 plate reader and analyzed with GraphPad Prism 9. The control antibody data was fit to a four-parameter curve and the relative sera concentration calculated by fitting the corresponding absorbance to these parameters.

### Serum neutralization assays

Murine macrophage-like J774A.1 cells (ATCC) were cultured at 37°C and 5% CO_2_ in DMEM with 1 mM sodium pyruvate, 10% FBS, and pen/strep and scraped with a rubber spatula collect cells for subculture or assay preparation. Cytotoxicity assays were performed as described[35]. Briefly, J774A.1 cells were seeded in 96-well plates at 1x10^5^ cells per 100 μL media. The next day, sera (1:250 dilution), murine M1H5 antibody (20 μg/mL) diluted in media, or a media-only control, were pre-incubated with 0.5 μg/mL refolded ACT in unsupplemented DMEM for 30 minutes at room temperature. Cells were washed three times with PBS before adding 110μL antibody-toxin media or 100μL media for lysis control wells. The plate was incubated at 37°C, 5% CO_2_ for 2 hours, with the addition of 10μL lysis reagent (Promega CytoTox96) to control wells after 75 minutes. The plate was centrifuged at 250xg for 5 minutes and the lactate dehydrogenase released measured by transferring 40 mL of supernatant to a fresh low binding 96-well plate (Corning) and adding an equal volume of CytoTox96 reagent. The reaction was incubated in the dark for 30 min before quenching with 40 mL 1 M acetic acid. The absorbance at 490 nm was measured and the % lysis for each well determined as follows: 100%*[A_490_ (sample well)—A_490_ (untreated cell control)]/[A_490_ (lysis control)—A_490_ (untreated cell control)].

## Supporting information

S1 Table Cap fusion designs. (DOCX)

S2 Table Crystallographic data collection and refinement statistics. (DOCX)

## References

[1] de Melker HE, Schellekens JF, Neppelenbroek SE, Mooi FR, Rumke HC, Conyn-van Spaendonck MA. Reemergence of pertussis in the highly vaccinated population of the Netherlands: observations on surveillance data. Emerg Infect Dis. 2000;6(4):348–57. doi: 10.3201/eid0604.000404 10905967 PMC2640897

[2] Rendi-Wagner P, Tobias J, Moerman L, Goren S, Bassal R, Green M, et al. The seroepidemiology of Bordetella pertussis in Israel—Estimate of incidence of infection. Vaccine. 2010;28(19):3285–90. doi: 10.1016/j.vaccine.2010.02.104 20226250

[3] De Serres G, Boulianne N, Douville Fradet M, Duval B. Pertussis in Quebec: ongoing epidemic since the late 1980s. Can Commun Dis Rep. 1995;21(5):45–8. 7757050

[4] Witt MA, Katz PH, Witt DJ. Unexpectedly limited durability of immunity following acellular pertussis vaccination in preadolescents in a North American outbreak. Clin Infect Dis. 2012;54(12):1730–5. doi: 10.1093/cid/cis287 22423127

[5] Christie CD, Marx ML, Marchant CD, Reising SF. The 1993 epidemic of pertussis in Cincinnati. Resurgence of disease in a highly immunized population of children. N Engl J Med. 1994;331(1):16–21. doi: 10.1056/NEJM199407073310104 8202096

[6] Baron S, Njamkepo E, Grimprel E, Begue P, Desenclos JC, Drucker J, et al. Epidemiology of pertussis in French hospitals in 1993 and 1994: thirty years after a routine use of vaccination. Pediatr Infect Dis J. 1998;17(5):412–8. doi: 10.1097/00006454-199805000-00013 9613656

[7] Halperin SA, Bortolussi R, MacLean D, Chisholm N. Persistence of pertussis in an immunized population: results of the Nova Scotia Enhanced Pertussis Surveillance Program. J Pediatr. 1989;115(5 Pt 1):686–93. 2809899 10.1016/s0022-3476(89)80643-2

[8] Tartof SY, Lewis M, Kenyon C, White K, Osborn A, Liko J, et al. Waning immunity to pertussis following 5 doses of DTaP. Pediatrics. 2013;131(4):e1047–52. doi: 10.1542/peds.2012-1928 23478868

[9] Koepke R, Eickhoff JC, Ayele RA, Petit AB, Schauer SL, Hopfensperger DJ, et al. Estimating the effectiveness of tetanus-diphtheria-acellular pertussis vaccine (Tdap) for preventing pertussis: evidence of rapidly waning immunity and difference in effectiveness by Tdap brand. J Infect Dis. 2014;210(6):942–53. doi: 10.1093/infdis/jiu322 24903664

[10] Lavine JS, Bjornstad ON, de Blasio BF, Storsaeter J. Short-lived immunity against pertussis, age-specific routes of transmission, and the utility of a teenage booster vaccine. Vaccine. 2012;30(3):544–51. doi: 10.1016/j.vaccine.2011.11.065 22119924 PMC3246080

[11] Sheridan SL, Frith K, Snelling TL, Grimwood K, McIntyre PB, Lambert SB. Waning vaccine immunity in teenagers primed with whole cell and acellular pertussis vaccine: recent epidemiology. Expert Rev Vaccines. 2014;13(9):1081–106. doi: 10.1586/14760584.2014.944167 25093268

[12] Warfel JM, Zimmerman LI, Merkel TJ. Acellular pertussis vaccines protect against disease but fail to prevent infection and transmission in a nonhuman primate model. Proc Natl Acad Sci U S A. 2014;111(2):787–92. doi: 10.1073/pnas.1314688110 24277828 PMC3896208

[13] Bouchez V, Brun D, Cantinelli T, Dore G, Njamkepo E, Guiso N. First report and detailed characterization of B. pertussis isolates not expressing Pertussis Toxin or Pertactin. Vaccine. 2009;27(43):6034–41. doi: 10.1016/j.vaccine.2009.07.074 19666155

[14] Hegerle N, Guiso N. Bordetella pertussis and pertactin-deficient clinical isolates: lessons for pertussis vaccines. Expert Rev Vaccines. 2014;13(9):1135–46. doi: 10.1586/14760584.2014.932254 24953157

[15] Bouchez V, Hegerle N, Strati F, Njamkepo E, Guiso N. New Data on Vaccine Antigen Deficient Bordetella pertussis Isolates. Vaccines (Basel). 2015;3(3):751–70. doi: 10.3390/vaccines3030751 26389958 PMC4586476

[16] Martin SW, Pawloski L, Williams M, Weening K, DeBolt C, Qin X, et al. Pertactin-negative Bordetella pertussis strains: evidence for a possible selective advantage. Clin Infect Dis. 2015;60(2):223–7. doi: 10.1093/cid/ciu788 25301209

[17] Hegerle N, Paris AS, Brun D, Dore G, Njamkepo E, Guillot S, et al. Evolution of French Bordetella pertussis and Bordetella parapertussis isolates: increase of Bordetellae not expressing pertactin. Clin Microbiol Infect. 2012;18(9):E340–6. doi: 10.1111/j.1469-0691.2012.03925.x 22717007

[18] Mooi FR, van Loo IH, van Gent M, He Q, Bart MJ, Heuvelman KJ, et al. Bordetella pertussis strains with increased toxin production associated with pertussis resurgence. Emerg Infect Dis. 2009;15(8):1206–13. doi: 10.3201/eid1508.081511 19751581 PMC2815961

[19] Weiss AA, Hewlett EL, Myers GA, Falkow S. Pertussis toxin and extracytoplasmic adenylate cyclase as virulence factors of Bordetella pertussis. J Infect Dis. 1984;150(2):219–22. doi: 10.1093/infdis/150.2.219 6088647

[20] Goodwin MS, Weiss AA. Adenylate cyclase toxin is critical for colonization and pertussis toxin is critical for lethal infection by Bordetella pertussis in infant mice. Infect Immun. 1990;58(10):3445–7. doi: 10.1128/iai.58.10.3445-3447.1990 2401570 PMC313675

[21] Guiso N, Rocancourt M, Szatanik M, Alonso JM. Bordetella adenylate cyclase is a virulence associated factor and an immunoprotective antigen. Microb Pathog. 1989;7(5):373–80. doi: 10.1016/0882-4010(89)90040-5 2622329

[22] Linhartova I, Bumba L, Masin J, Basler M, Osicka R, Kamanova J, et al. RTX proteins: a highly diverse family secreted by a common mechanism. FEMS Microbiol Rev. 2010;34(6):1076–112. doi: 10.1111/j.1574-6976.2010.00231.x 20528947 PMC3034196

[23] Welch RA. Pore-forming cytolysins of gram-negative bacteria. Mol Microbiol. 1991;5(3):521–8. doi: 10.1111/j.1365-2958.1991.tb00723.x 2046545

[24] Baumann U. Crystal structure of the 50 kDa metallo protease from Serratia marcescens. J Mol Biol. 1994;242(3):244–51. doi: 10.1006/jmbi.1994.1576 8089845

[25] Angkawidjaja C, Kanaya S. Family I.3 lipase: bacterial lipases secreted by the type I secretion system. Cell Mol Life Sci. 2006;63(23):2804–17. doi: 10.1007/s00018-006-6172-x 17103114 PMC11136109

[26] Baumann U, Wu S, Flaherty KM, McKay DB. Three-dimensional structure of the alkaline protease of Pseudomonas aeruginosa: a two-domain protein with a calcium binding parallel beta roll motif. EMBO J. 1993;12(9):3357–64. 8253063 10.1002/j.1460-2075.1993.tb06009.x PMC413609

[27] Garnham CP, Campbell RL, Davies PL. Anchored clathrate waters bind antifreeze proteins to ice. Proc Natl Acad Sci U S A. 2011;108(18):7363–7. doi: 10.1073/pnas.1100429108 21482800 PMC3088597

[28] Guo S, Stevens CA, Vance TDR, Olijve LLC, Graham LA, Campbell RL, et al. Structure of a 1.5-MDa adhesin that binds its Antarctic bacterium to diatoms and ice. Sci Adv. 2017;3(8):e1701440. doi: 10.1126/sciadv.1701440 28808685 PMC5550230

[29] Chenal A, Guijarro JI, Raynal B, Delepierre M, Ladant D. RTX calcium binding motifs are intrinsically disordered in the absence of calcium: implication for protein secretion. J Biol Chem. 2009;284(3):1781–9. doi: 10.1074/jbc.M807312200 19015266

[30] Bumba L, Masin J, Macek P, Wald T, Motlova L, Bibova I, et al. Calcium-Driven Folding of RTX Domain beta-Rolls Ratchets Translocation of RTX Proteins through Type I Secretion Ducts. Mol Cell. 2016;62(1):47–62. doi: 10.1016/j.molcel.2016.03.018 27058787

[31] Bauche C, Chenal A, Knapp O, Bodenreider C, Benz R, Chaffotte A, et al. Structural and functional characterization of an essential RTX subdomain of Bordetella pertussis adenylate cyclase toxin. J Biol Chem. 2006;281(25):16914–26. doi: 10.1074/jbc.M601594200 16627468

[32] Blenner MA, Shur O, Szilvay GR, Cropek DM, Banta S. Calcium-induced folding of a beta roll motif requires C-terminal entropic stabilization. J Mol Biol. 2010;400(2):244–56. doi: 10.1016/j.jmb.2010.04.056 20438736

[33] Guermonprez P, Khelef N, Blouin E, Rieu P, Ricciardi-Castagnoli P, Guiso N, et al. The adenylate cyclase toxin of Bordetella pertussis binds to target cells via the alpha(M)beta(2) integrin (CD11b/CD18). J Exp Med. 2001;193(9):1035–44. doi: 10.1084/jem.193.9.1035 11342588 PMC2193436

[34] Osicka R, Osickova A, Hasan S, Bumba L, Cerny J, Sebo P. Bordetella adenylate cyclase toxin is a unique ligand of the integrin complement receptor 3. Elife. 2015;4:e10766. doi: 10.7554/eLife.10766 26650353 PMC4755762

[35] Wang X, Gray MC, Hewlett EL, Maynard JA. The Bordetella adenylate cyclase repeat-in-toxin (RTX) domain is immunodominant and elicits neutralizing antibodies. J Biol Chem. 2015;290(38):23025. doi: 10.1074/jbc.A114.585281 26386047 PMC4645631

[36] Wang X, Stapleton JA, Klesmith JR, Hewlett EL, Whitehead TA, Maynard JA. Fine Epitope Mapping of Two Antibodies Neutralizing the Bordetella Adenylate Cyclase Toxin. Biochemistry. 2017;56(9):1324–36. doi: 10.1021/acs.biochem.6b01163 28177609 PMC5568097

[37] Motlova L, Klimova N, Fiser R, Sebo P, Bumba L. Continuous Assembly of beta-Roll Structures Is Implicated in the Type I-Dependent Secretion of Large Repeat-in-Toxins (RTX) Proteins. J Mol Biol. 2020;432(20):5696–710. doi: 10.1016/j.jmb.2020.08.020 32860773

[38] Parmeggiani F, Huang PS. Designing repeat proteins: a modular approach to protein design. Curr Opin Struct Biol. 2017;45:116–23. doi: 10.1016/j.sbi.2017.02.001 28267654

[39] Espinosa-Vinals CA, Masin J, Holubova J, Stanek O, Jurnecka D, Osicka R, et al. Almost half of the RTX domain is dispensable for complement receptor 3 binding and cell-invasive activity of the adenylate cyclase toxin. J Biol Chem. 2021:100833. doi: 10.1016/j.jbc.2021.100833 34051233 PMC8214218

[40] Kohl A, Binz HK, Forrer P, Stumpp MT, Pluckthun A, Grutter MG. Designed to be stable: crystal structure of a consensus ankyrin repeat protein. Proc Natl Acad Sci U S A. 2003;100(4):1700–5. doi: 10.1073/pnas.0337680100 12566564 PMC149896

[41] Main ER, Xiong Y, Cocco MJ, D’Andrea L, Regan L. Design of stable alpha-helical arrays from an idealized TPR motif. Structure. 2003;11(5):497–508. doi: 10.1016/s0969-2126(03)00076-5 12737816

[42] Guiso N, Szatanik M, Rocancourt M. Protective activity of Bordetella adenylate cyclase-hemolysin against bacterial colonization. Microb Pathog. 1991;11(6):423–31. doi: 10.1016/0882-4010(91)90038-c 1795632

[43] Cheung GY, Xing D, Prior S, Corbel MJ, Parton R, Coote JG. Effect of different forms of adenylate cyclase toxin of Bordetella pertussis on protection afforded by an acellular pertussis vaccine in a murine model. Infect Immun. 2006;74(12):6797–805. doi: 10.1128/IAI.01104-06 16982827 PMC1698075

[44] Holubova J, Stanek O, Brazdilova L, Masin J, Bumba L, Gorringe AR, et al. Acellular Pertussis Vaccine Inhibits Bordetella pertussis Clearance from the Nasal Mucosa of Mice. Vaccines (Basel). 2020;8(4). doi: 10.3390/vaccines8040695 33228165 PMC7711433

[45] Battye TG, Kontogiannis L, Johnson O, Powell HR, Leslie AG. iMOSFLM: a new graphical interface for diffraction-image processing with MOSFLM. Acta Crystallogr D Biol Crystallogr. 2011;67(Pt 4):271–81. doi: 10.1107/S0907444910048675 21460445 PMC3069742

[46] Evans PR, Murshudov GN. How good are my data and what is the resolution? Acta Crystallogr D Biol Crystallogr. 2013;69(Pt 7):1204–14. doi: 10.1107/S0907444913000061 23793146 PMC3689523

[47] McCoy AJ, Grosse-Kunstleve RW, Adams PD, Winn MD, Storoni LC, Read RJ. Phaser crystallographic software. J Appl Crystallogr. 2007;40(Pt 4):658–74. doi: 10.1107/S0021889807021206 19461840 PMC2483472

[48] Emsley P, Cowtan K. Coot: model-building tools for molecular graphics. Acta Crystallogr D Biol Crystallogr. 2004;60(Pt 12 Pt 1):2126–32. doi: 10.1107/S0907444904019158 15572765

[49] Adams PD, Grosse-Kunstleve RW, Hung LW, Ioerger TR, McCoy AJ, Moriarty NW, et al. PHENIX: building new software for automated crystallographic structure determination. Acta Crystallogr D Biol Crystallogr. 2002;58(Pt 11):1948–54. doi: 10.1107/s0907444902016657 12393927

[50] Croll TI. ISOLDE: a physically realistic environment for model building into low-resolution electron-density maps. Acta Crystallogr D Struct Biol. 2018;74(Pt 6):519–30. doi: 10.1107/S2059798318002425 29872003 PMC6096486

