# Supplementary material for: Structural basis for antibody binding to adenylate cyclase toxin reveals RTX linkers as neutralization-sensitive epitopes
Source: PLoS Pathog. 2021 Sep 21;17(9):e1009920. doi: 10.1371/journal.ppat.1009920 (PMC8486122; doi:10.1371/journal.ppat.1009920)
Supplement: S1 Table — (DOCX) [file ppat.1009920.s001.docx]

**Table S1. Cap fusion designs.**

| **Cap fusion variant** | **N-terminal amino acids** | **C-terminal amino acids** |
| --- | --- | --- |
| F1 | 1007-1350 | 1610-1706 |
| F2 | 1007-1344 | 1604-1706 |
| F3 | 1007-1330 | 1601-1706 |
| F4 | 1007-1324 | 1594-1706 |
| F5 (“123cap”) | 1007-1350 | 1589-1706 |
