# Supplementary material for: Structural basis for antibody binding to adenylate cyclase toxin reveals RTX linkers as neutralization-sensitive epitopes
Source: PLoS Pathog. 2021 Sep 21;17(9):e1009920. doi: 10.1371/journal.ppat.1009920 (PMC8486122; doi:10.1371/journal.ppat.1009920)
Supplement: S2 Table — (DOCX) [file ppat.1009920.s002.docx]

Table S2. Crystallographic data collection and refinement statistics.

|  | 123cap+M2B10 Fab+M1H5 Fab |
| --- | --- |
| **PDB ID**  **Data collection** | 7RAH |
| Space group  Wavelength (Å) | *P*2_1_2_1_2_1_  0.979 |
| Cell dimensions |  |
| *a, b, c* (Å) | 65.6, 117.0, 254.7 |
| α=β=γ (°) | 90 |
| Resolution (Å) | 86.17-2.60 (2.67-2.60) |
| *R*_merge_ | 0.069 (0.739) |
| *I* / σ*I*  CC_1/2_ | 7.4 (2.2)  0.983 (0.771) |
| Completeness (%) | 100 (100) |
| Redundancy  Total reflections  Unique reflections | 5.7 (5.9)  350,884 (26,574)  61,228 (4,468) |
| **Refinement** |  |
| Resolution (Å) | 55.81-2.60 (2.64-2.60) |
| Unique reflections | 61,093 (2,735) |
| *R*_work_ / *R*_free_ (%) | 19.5/21.8 |
| No. atoms | 9,183 |
| Protein | 8,972 |
| Water  Ca^2+^  PO_4_^3-^ | 188  18  5 |
| *B*-factors (Å^2^) |  |
| Protein | 65.0 |
| Water  Ca^2+^/ PO_4_^3-^ | 55.9  58.9 |
| R.m.s. deviations  Bond lengths (Å) | 0.002 |
| Bond angles (°)  Ramachandran (%)  Favored  Allowed  Outliers | 0.55  97.1  2.9  0.0 |

Data were collected from one crystal. *Values in parentheses are for highest-resolution shell.
